# The prevalence and time trend of asthma in Albanian children in 2011 – Alb ISAAC

**DOI:** 10.1186/2045-7022-5-S2-P8

**Published:** 2015-03-23

**Authors:** Eris Mesonjesi, Erjola Piluri Ziu, Ramyani Gupta, David Strachan, Alfred Priftanji

**Affiliations:** 1 University Hospital Center "Mother Teresa", TIRANA, Albania; 2 American Hospital Tirana, Department of Allergology and Clinical Immunology, TIRANA, Albania; 3 St George's University of London, Population Health Research Institute, London, UK; 4 University Hospital Center "Mother Teresa", Department of Allergology and Clinical Immunology, TIRANA, Albania

## Background

A large epidemiological study, Alb ISAAC was conducted in Tirana in 2011-2012.

## Aims

The aims were both cross-sectional and longitudinal. Cross-sectionally (in 2011) we aimed to describe the prevalence and severity of asthma, rhinoconjunctivitis and atopic dermatitis in children living in Tirana and to estimate the risk factors for these conditions. Longitudinally (over the past 12-16 years) we aimed to examine time trends in the prevalence and severity of these diseases in Tirana from 1995 to 2011; and also trends in the risk factors for developing asthma from 1999 to 2011.

## Methods

The Alb-ISAAC 2011 study is part of a series of population surveys carried out in Tirana, using the internationally recognised methodology of the ISAAC survey. Symptom questionnaires for asthma, rhinitis and examination for visible flexural dermatitis were completed for a total of 9280 children. Additionally, in the 10-11 age-groups, risk factor questionnaires were completed for 3204 children and allergen skin prick tests (SPT) were performed on 1093 of these. Response rates exceeded 85% in all parts of the survey.

## Results

The prevalence of reported asthma ever in 1995 and 2011 are respectively for the 6-7 years old (yo) 3.1% and 2.8% (OR= 0.9, 95% CI 0.7-1.2), for 13-14 yo: 1.6% and 4.4%(OR= 2.9, 95% CI 2.0-4.0), and for 10-11yo: 2.7% and 3.0% (OR= 1.14, 95% CI 0.74-1.75). The results for wheeze in last 12 months: 6-7 years old (yo) 7.6% and 6.1% (OR= 0.8, 95% CI 0.6-1.0), for 13-14 yo: 2.6% and 2.9% (OR= 1.1, 95% CI 0.8-1.8), and for 10-11yo: 4.4% and 4.6% (OR= 1.07, 95% CI 0.76-1.50). The prevalence of sensitization for any allergen was 24.3 %. A lifetime history of asthma became more common in the older age groups. No significant changes in the severity of asthma were found. Wheeze in the last year and asthma ever were consistently more common among skin prick tests (SPT)+ children in both surveys 1999 and 2011, but the indicators of more severe wheezing showed a variable pattern.

## Conclusions

Despite substantial changes in our lifestyle and home environment over the past 12-16 years, Albanian children remain at low risk of asthma symptoms compared to other ISAAC centres in Europe. In contrast, the prevalence of allergic sensitization as measured by allergen skin prick tests increased significantly, comparable with the English speaking countries but is not clearly translated in more allergic symptoms or more diseases.

